# The Impact of Ursodeoxycholic Acid on Maternal Cardiac Function in Women with Gestational Diabetes Mellitus: A Randomized Controlled Study (GUARDS Trial)

**DOI:** 10.3390/jcm15020786

**Published:** 2026-01-19

**Authors:** Ana María Company Calabuig, Jose Eliseo Blanco-Carnero, Christos Chatzakis, Catherine Williamson, Kypros H. Nicolaides, Catalina De Paco Matallana, Marietta Charakida

**Affiliations:** 1 Department of Obstetrics and Gynecology, Hospital Clínico Universitario Virgen de la Arrixaca, El Palmar, 30120 Murcia, Spain; anamariacompanycalabuig@gmail.com (A.M.C.C.); jeblancoc@gmail.com (J.E.B.-C.); 2 Faculty of Medicine, Universidad de Murcia, 30107 Murcia, Spain; 3 Biomedical Research Institute of Murcia Pascual Parrilla—IMIB, 30120 Murcia, Spain; 4 Harris Birthright Research Centre for Fetal Medicine, King’s College, London SE5 8BB, UK; cchatzakis@gmail.com (C.C.); charakidadoc@googlemail.com (M.C.); 5 Institute of Reproductive and Developmental Biology, Department of Metabolism, Digestion & Reproduction, Imperial College London, London W12 0NN, UK; catherine.williamson@imperial.ac.uk; 6 School of Biomedical Engineering and Imaging Sciences, King’s College London, London SE1 7EH, UK

**Keywords:** gestational diabetes mellitus, ursodeoxycholic acid, maternal cardiac function, echocardiography, pregnancy

## Abstract

**Background**: Gestational diabetes mellitus (GDM) is associated with metabolic disturbance and subclinical cardiovascular changes during pregnancy and after birth. Optimal glycaemic control remains challenging for many patients despite existing management strategies. Ursodeoxycholic acid (UDCA) has shown potential metabolic effects, including enhanced insulin sensitivity and anti-inflammatory effects. Previously, we demonstrated that UDCA improves glycaemic control in women achieving higher circulating UDCA concentrations; however, its effect on maternal cardiac function remains unknown. The objective was to evaluate whether treatment with UDCA compared with placebo is associated with differences in maternal cardiac function in pregnancies complicated by GDM. **Methods**: In this randomized, placebo-controlled trial, 113 women with GDM were recruited, with 56 allocated to UDCA and 57 to placebo (IMIB-GU-2019-02, registration date: 17 June 2020; first participant enrolled: 3 March 2021). After measurement of maternal blood UDCA levels, 43 participants in the treatment group with levels ≥ 0.5 μmol/L were included in a per-protocol analysis. Participants had cardiac assessments at baseline, in the late third trimester (36 weeks) and postpartum. Detailed left ventricular systolic and diastolic functional indices were assessed using conventional pulse and tissue Doppler indices as well as strain imaging. Right ventricular systolic function was also assessed. **Results**: Baseline maternal characteristics and cardiac functional indices were comparable between the UDCA and placebo groups. In the third trimester, women treated with UDCA showed more negative left atrial strain during atrial contraction (LASct_AC) compared with placebo (*p* = 0.016), while no significant between-group differences were observed in conventional left ventricular systolic or diastolic parameters. In the postpartum period, UDCA treatment was associated with higher left atrial reservoir function, reflected by increased LASr_ED (*p* = 0.041) and LASr_AC (*p* = 0.036), as well as more negative left atrial conduit strain at end-diastole (LAScd_ED; *p* = 0.043). No consistent differences were observed in left ventricular systolic function, haemodynamic indices, or right ventricular functional parameters between the two groups. **Conclusions**: These findings are associated with small and time-dependent differences in reducing atrial dysfunction and improving cardiac efficiency during late pregnancy and postpartum. However, given the lack of long-term follow-up, further research is needed to determine the long-term cardiovascular relevance of UDCA in this population.

## 1. Introduction

Gestational diabetes mellitus (GDM) is a pregnancy condition characterised by glucose intolerance that can lead to adverse outcomes for both the mother and her children [1,2]. Beyond pregnancy, GDM is associated with a higher long-term maternal cardiometabolic risk. Large studies show that women with a history of GDM exhibit an increased risk of developing type 2 diabetes and cardiovascular disease, highlighting the persistent nature of the metabolic changes triggered during pregnancy [1,2,3,4,5]. This suggests that GDM represents not only a transient pregnancy complication but also an early clinical marker of long-term cardiovascular vulnerability.

Our group and others have demonstrated that during pregnancy, women with GDM have mild cardiovascular changes, including a reduction in left ventricular systolic function and an increase in arterial stiffness, compared to those with uncomplicated pregnancies [3,4]. In addition, these changes persist for at least 6 months postpartum [5]. Such subclinical impairments in cardiac performance may have implications for both short-term haemodynamic adaptation and long-term maternal cardiovascular health. Despite increasing recognition of these alterations, few interventions have been investigated with the aim of improving cardiac function during pregnancies affected by GDM.

Pregnancy is characterised by significant metabolic adaptations, including progressive insulin resistance mediated by placental hormones such as placental lactogen, progesterone, cortisol, and placental growth factor. In women who develop gestational diabetes mellitus, these physiological changes exceed adaptive capacity, leading to hyperglycaemia, dyslipidaemia, low-grade inflammation, and endothelial dysfunction. These metabolic alterations impose extra demands on the maternal cardiovascular system, which may lead to subclinical cardiac remodelling [6,7].

In the fetus, exposure to a hyperglycaemic intrauterine environment is associated with altered myocardial growth, diastolic dysfunction, and reduced cardiac efficiency, influenced by fetal hyperinsulinemia, oxidative stress, and metabolic programming. These cardiovascular changes in both the mother and the fetus indicate a need for new interventions that could positively impact maternal cardiac adaptation and the intrauterine environment in pregnancies complicated by GDM [8].

Given the metabolic and cardiovascular changes associated with GDM, there is a growing interest in potential therapeutic agents that may improve both glycemic control and cardiovascular health during pregnancy. Ursodeoxycholic acid (UDCA) is a hydrophilic bile acid that is mostly used in the treatment of cholestatic liver diseases, including intrahepatic cholestasis of pregnancy (ICP) and primary biliary cholangitis. UDCA has demonstrated multiple beneficial effects, including anti-inflammatory, antioxidative, and cytoprotective properties. Recent studies suggest that UDCA may also play a role in improving insulin sensitivity, lipid metabolism, and endothelial function, making it a promising candidate for managing metabolic disorders such as GDM [9].

We have previously shown that total serum bile acid levels in serum are positively correlated with serum glucose and fasting insulin concentrations in women with GDM [10], indicating that a treatment such as UDCA that normalises bile acid profiles may improve clinical features of GDM. Beyond its hepatic benefits, UDCA has been shown to exert cardioprotective effects by reducing oxidative stress, modulating inflammatory pathways, and enhancing mitochondrial function [11]. These mechanisms are particularly relevant in GDM, where metabolic dysregulation can negatively impact maternal cardiac function. However, whether these biological effects translate into measurable differences in maternal cardiac function during pregnancy remains uncertain. The potential of UDCA to reduce cardiac strain in pregnant women with GDM remains unknown.

The aim of this sub-study within the GUARDS trial was to assess the effect of UDCA compared with placebo on maternal cardiac function in pregnancies complicated by GDM.

## 2. Materials and Methods

### 2.1. Study Design and Participants

This prospective study was carried out as part of the GUARDS trial (a randomized, placebo-controlled, double-blind trial designed to evaluate the effect of UDCA’s impact on glycemic control in women with gestational diabetes mellitus) [12]. This trial was registered at the Spanish Clinical Trials Registry (https://reec.aemps.es/reec/public/web.html, accessed on 17 June 2020 (IMIB-GU-2019-02); registration date: 17 June 2020; first participant enrolled on 3 March 2021). This study was carried out at the Clinical University Hospital “Virgen de la Arrixaca” in Murcia, Spain, and received approval from the relevant national research ethics committees and competent regulatory authorities in Murcia.

The primary endpoint of the GUARDS trial was maternal glycaemic control in late pregnancy. Maternal cardiac assessment constituted a prespecified secondary objective, designed to explore potential associations between UDCA treatment and maternal cardiac structure and function. The trial was not independently powered for cardiac endpoints, and no formal sample size calculation was performed for echocardiographic outcomes.

Women with an abnormal oral glucose tolerance test (OGTT) screen were invited to participate between 24^+0^ and 30^+6^ weeks’ gestation. In Spain, the diagnosis of GDM requires a two-step strategy: The first step is the O’Sullivan Test–conducted between 24 and 28 weeks of gestation, in which a plasma glucose concentration ≥ 140 mg/dL (7.8 mmol/L) one hour after a 50 g oral glucose load is considered abnormal. Women with a positive screening test subsequently underwent a diagnostic 100 g, 3 h OGTT after an overnight fast. GDM was confirmed when at least two of the following thresholds were met: fasting glucose ≥ 95 mg/dL (5.3 mmol/L), 1-h glucose ≥ 180 mg/dL (10.0 mmol/L), 2-h glucose ≥ 155 mg/dL (8.6 mmol/L), and 3-h glucose ≥ 140 mg/dL (7.8 mmol/L) [1,2].

Inclusion criteria included GDM diagnosed at 24–28 weeks’ gestation, planned antenatal care at the same centre, singleton pregnancy, and the ability to provide informed and written consent. Exclusion criteria were age < 18 years, diabetes type 1 and type 2, significant pre-pregnancy medical conditions, and major medical or obstetric complications.

Women with isolated insulin resistance or mildly elevated glycaemic values that did not meet GDM diagnostic criteria were not included, as the trial was designed to evaluate a clearly defined GDM population.

### 2.2. Sample Size Estimation

Initial sample size calculations for the GUARDS trial indicated that 204 participants would be sufficient to provide 90% power to detect a significant reduction in maternal fasting glucose at 36 weeks (the primary outcome of the GUARDS trial), allowing for an anticipated withdrawal rate of 40%. Due to COVID-related delays and limited availability of the placebo, updated calculations were performed. By the time the investigational product expired, 113 participants had been recruited, with 5 withdrawing. Revised estimates showed that 50 women per group would provide 82% power and 55 women per group would provide 85% power to detect the primary outcome at a two-sided significance level of *p* < 0.05.

### 2.3. Randomization and Study Procedures

After written informed consent, eligible women were randomly assigned to receive UDCA 500 mg twice daily or placebo until delivery according to the GUARDS study [12], and both participants and investigators were blinded to treatment allocation. In the present study, women who were allocated either to the UDCA or placebo group agreed to have a cardiac scan on the day of randomization and before starting medication, at 35–37 weeks and 3 months postpartum.

As part of the original GUARDS trial protocol, circulating UDCA concentrations were measured using ultra-performance liquid chromatography–tandem mass spectrometry (UPLC–MS/MS). In the primary GUARDS analysis, an unplanned secondary analysis explored the correlation between fasting blood glucose concentrations and circulating UDCA levels, which led to the identification of a threshold of 0.5 μmol/L to define supraphysiological UDCA exposure. This cut-off was chosen based on the distribution of measured UDCA concentrations in participants who received placebo, in whom circulating levels consistently remained below this value [12].

In this cardiac sub-study, the same threshold (≥0.5 μmol/L) was used to determine biologically significant exposure to the study drug and to minimize a misclassification of participants with negligible or absent systemic UDCA absorption. Accordingly, cardiac analyses were conducted as a per-protocol analysis, limited to women allocated to the UDCA group with measurable circulating UDCA concentrations ≥ 0.5 μmol/L. This approach represents a deviation from a strict intention-to-treat analysis and may introduce selection bias; therefore, the results should be interpreted with caution.

**Figure 1:**
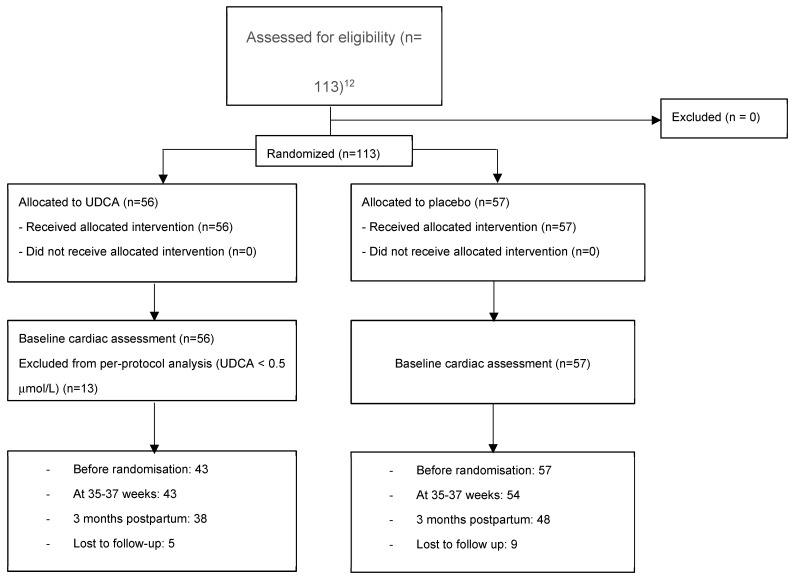
CONSORT flow diagram showing participant enrolment, randomization, allocation, follow-up, and analysis for the GUARDS trial cardiac sub-study. Cardiac analyses in the UDCA group were conducted as a per-protocol analysis based on circulating UDCA concentrations [13].

Participant enrolment, randomization, per-protocol definition, follow-up, and analysis for the cardiac sub-study are summarised in Figure 1.

### 2.4. Maternal Characteristics

Maternal demographic and clinical characteristics were recorded, including age, ethnic origin (white, black, asian, or mixed), method of conception (spontaneous or assisted, including in vitro fertilisation or ovulation induction), smoking, and parity (parous or nulliparous in the absence of a previous delivery at ≥24 weeks’ gestation). At each clinic visit, maternal weight and height were measured, and body mass index was calculated.

### 2.5. Maternal Cardiac Function Analysis

Maternal echocardiography was performed using the X5-1 transducer with an EPIQ 7G or EPIQ Elite ultrasound machine (Philips, Bothell, WA, USA), according to the European Association of Cardiovascular Imaging/American Society of Echocardiography guidelines [14]. Measurements of the left ventricle and atrium were obtained in the standard four-chamber apical view, whereas measurements of the right ventricle were performed at a 45° angle. The clips were exported in the original frame rate to an external hard drive and transferred for offline analysis using the QLAB Advanced Quantification software QLAB 10.x (Philips). Using 3D echocardiography, the ejection fractions of the left and right ventricles were calculated, as well as left ventricular mass. Left ventricular mass was indexed to body surface area. Longitudinal right ventricular functional assessment was also performed by calculating tricuspid annular plane systolic excursion (TAPSE) and fractional area change. Speckle-tracking echocardiography was used to assess global longitudinal strain (GLS) of the left and right ventricles, as described previously [15]. Starting from the initial end-systolic contour, the software used an established speckle-tracking algorithm to automatically detect the endocardial borders in all frames of the selected cardiac cycle. Measurements were performed at 60–70 frames per second. Left atrial area and volume were calculated manually in end systole from the four-chamber apical view in 2D, as described previously [11]. Left atrial strain measurements were performed in accordance with the European Association of Cardiovascular Imaging guidelines [16]. Investigators were blinded to treatment allocation.

Baseline cardiac assessment was performed on the day of randomization and prior to initiation of UDCA or insulin therapy. Follow-up echocardiographic examinations were conducted at predefined gestational windows (35–37 weeks) and 3 months postpartum, independent of the timing of OGTT or changes in glycemic management.

### 2.6. Statistical Analysis

Continuous variables were reported as mean ± SD if their distribution was normal or as median (interquartile range) if their distribution was non-normal. Normality of the distribution was assessed using the Kolmogorov–Smirnov test. Categorical variables were presented as n (%). Individual comparison between the placebo and treatment groups in the late third trimester and postpartum was performed using a *t*-test. *P*-values are presented for the direct two-way comparisons between groups. *p* < 0.05 was considered statistically significant.

Linear mixed-effects models were used to evaluate the association between treatment group, time, and their interaction on maternal cardiac indices. Time was modelled as a categorical variable (baseline, 36 weeks, postpartum), and a random intercept for each participant was included to account for within-subject correlation. Models were adjusted for prespecified maternal characteristics. Analyses were performed using R software R 4.5.1 [17].

Missing data were handled within the mixed-effects framework under the assumption of missing at random. Model assumptions were assessed using visual inspection of residuals. Given the exploratory nature of the cardiac outcomes, no correction for multiple comparisons was applied.

## 3. Results

### 3.1. Participant Characteristics

Before randomization, there were no significant differences between groups with regard to participant characteristics and cardiac functional indices. Individual comparisons between the placebo and treatment groups are shown in Table 1 and Table 2. Comprehensive baseline echocardiographic parameters are provided in Supplementary Table S1.

### 3.2. Comparison of Cardiac Indices During the Study

Individual comparisons between the treatment and placebo groups at 36 weeks’ gestation and postpartum did not show significant differences in the left ventricular diastolic or systolic indices, hemodynamic parameters, or right ventricular function (Table 3). Detailed unadjusted comparisons for additional ventricular, hemodynamic, and structural indices at these time points are provided in Supplementary Table S2.

Multivariable longitudinal analysis using mixed-effects models to account for differences in time and treatment indicated that left atrial functional indices and left ventricular systolic indices improved over time in the treatment group. Specifically, there was a statistically significant interaction for left atrial contractile strain during atrial contraction (LASct_AC) in the late third trimester (−3.22, 95% CI −6.06 to −0.37; *p* = 0.031), reflecting a reduction over time in the UDCA group, while no significant temporal change was observed in the placebo group. This interaction was not maintained in the postpartum period (*p* = 0.372).

To evaluate temporal changes in cardiac function, longitudinal mixed-effects models incorporating time, treatment group, and treatment-by-time interaction terms were employed. Corresponding model estimates are presented in Supplementary Table S3.

A significant interaction between treatment and time was also observed for septal mitral annular systolic velocity (MV_s_septal) in the late third trimester (0.75, 95% CI 0.01 to 1.48; *p* = 0.039), indicating a temporal increase in the UDCA group. However, this effect did not persist in the postpartum period (*p* = 0.795). No overall time effect was observed in the placebo group.

In the postpartum period, significant treatment-by-time interactions were identified for left atrial reservoir strain at end-diastole (LASr_ED; 10.69, 95% CI 2.90 to 18.48; *p* = 0.007) and during atrial contraction (LASr_AC; 7.57, 95% CI 1.99 to 13.15; *p* = 0.008). In addition, left atrial conduit strain (LAScd_ED; −7.03, 95% CI −12.63 to −1.42; *p* = 0.014) and left atrial contractile strain at end-diastole (LASct_ED; −4.40, 95% CI −8.23 to −0.57; *p* = 0.002) also demonstrated significant treatment-by-time interactions, with changes observed over time in the UDCA group but not in the placebo group.

Among the longitudinal analyses, LASct_AC was the only parameter showing a statistically significant treatment-by-time interaction in the late third trimester (*p* = 0.031), whereas this interaction was not observed postpartum.

## 4. Discussion

### 4.1. Principal Findings

The results of this study suggest that UDCA treatment was associated with small and selective differences in maternal cardiac function in women with GDM, particularly involving indices of left atrial function during late pregnancy and the postpartum period. These differences were detected mainly through longitudinal mixed-effects modelling, and were not consistently observed in unadjusted cross-sectional comparisons, suggesting potential alterations in cardiac functional trajectories over time rather than clear treatment-related effects.

The observed changes were small and confined to atrial functional parameters and should therefore be interpreted with caution. These findings enhance the understanding of maternal cardiovascular adaptation in GDM, but they do not support definitive conclusions regarding a cardioprotective effect of UDCA. Nevertheless, the use of strain imaging may provide a sensitive tool for detecting subtle, subclinical changes in maternal cardiac function in this population and warrants further investigation in adequately powered studies.

### 4.2. Results in the Context of What We Know

Several studies have examined maternal cardiovascular adaptation in pregnancies complicated by GDM, consistently demonstrating subtle but clinically relevant changes in cardiac function. Using conventional echocardiography and strain imaging, previous studies have shown reductions in left ventricular systolic performance, impaired diastolic relaxation, and altered atrial mechanics in women with GDM compared with those with uncomplicated pregnancies, changes that may persist into the postpartum period [18,19,20]. These findings support the concept that GDM represents a state of early cardiovascular vulnerability rather than a transient metabolic disturbance [1,2,3,4,5].

Our findings extend this body of literature by providing randomised, placebo-controlled evidence suggesting that pharmacological modulation with ursodeoxycholic acid may influence specific components of maternal cardiac function, particularly left atrial mechanics and systolic myocardial performance during late pregnancy and postpartum. Although the observed effects were small, the use of longitudinal mixed-effects modelling allowed detection of time-dependent changes that may not be apparent in cross-sectional analyses, aligning with previous strain-based studies in GDM populations [18,19,20].

Previous studies have shown that although hyperglycemia resolves after delivery for women with GDM, these women continue to have mild metabolic abnormalities and are at increased risk for adverse health outcomes [1,2]. For instance, a history of GDM has been associated with increased cardiovascular risk later in life [3,4,5]. However, it is uncertain whether the reported association between GDM and cardiovascular risk is the result of the hyperglycemic insult on the cardiovascular system during pregnancy or due to prolonged exposure to an adverse cardiovascular risk factor profile before, during and after pregnancy. Insulin resistance, obesity and elevated inflammatory markers have been reported in women who developed GDM several years after the index pregnancy, and these may increase their cardiovascular risks [1,2].

Our group and others have consistently reported mild cardiac functional changes with a reduction in left ventricular systolic and diastolic function in women with GDM, and, for the majority of women, these changes improve but do not normalise during the postpartum period [18,19,20]. In the current study, we had the opportunity to use a variety of traditional and novel echocardiographic indices, which enabled us to detect early changes in both systolic and diastolic ventricular mechanics. From the various indices, atrial strain imaging, which provides accurate assessment of left atrial and ventricular interplay, was more informative. In particular, left atrial strain during contraction (LASct_AC) was reduced in the treatment group, suggesting that UDCA improves atrial compliance and reduces atrial workload. This aspect may be particularly beneficial in GDM, where subclinical diastolic dysfunction and altered myocardial mechanics are common.

From additional echocardiographic indices, the temporary increase in mitral valve systolic velocity (MV_s_Vel_sep) in the late third trimester suggests that UDCA has a protective effect on maternal cardiac function against pregnancy-related haemodynamic stress. Although this effect did not persist postpartum, it raises the possibility that UDCA supports short-term systolic function during pregnancy, a period characterized by increased cardiac output demands.

In the postpartum period, we observed significant improvements in left atrial reservoir strain (LASr_ED) and contractile strain (LASr_AC) in the treatment group, suggesting that UDCA may confer sustained enhancements in atrial function beyond pregnancy. Additionally, reductions in conduit strain values (LAScd_ED and LASct_ED) may indicate improved ventricular compliance and more efficient passive filling. These changes were not observed in the placebo group, highlighting a treatment-specific effect. While not all echocardiographic indices showed persistent improvement postpartum, the sustained changes in select atrial functional markers raise the possibility that UDCA may contribute to favourable cardiac recovery after delivery.

### 4.3. Clinical and Research Implications

Our study suggests that UDCA may have a dynamic and time-dependent impact on maternal cardiac function by improving left atrial compliance, reducing atrial stiffness, and enhancing diastolic and systolic ventricular function. These results align with the hypothesis that UDCA may exert cardioprotective effects through its anti-inflammatory, metabolic, and endothelial-stabilising properties [8]. In addition, persistent cardiac benefits during postpartum suggest that short-term UDCA use may have long-term cardiovascular benefits by improving cardiac remodelling. Considering that women with GDM have an elevated risk of developing heart failure, hypertension, and atrial fibrillation later in life due to persistent metabolic and vascular dysfunction [3,5], our results are of particular importance and provide insights into a potential new role for UDCA in cardiovascular protection during and after pregnancy in women with GDM.

Our results provide insights into the potential of UDCA as a treatment for non-pregnant people with cardiac dysfunction. Studies of people with non-gestational cholestatic disorders, e.g., primary biliary cholangitis or biliary atresia, show abnormalities in cardiac function [21]. While there are no studies of the impact of UDCA on cardiac function in these disorders, UDCA has been shown to be antifibrotic by signalling through the membrane bile acid receptor TGR5 in models of cardiac fibrosis [11]. However, the significance of these findings in pregnant or postpartum women requires further investigation.

### 4.4. Strengths and Limitations

Strengths of this study are that it is the first prospective, placebo-controlled trial of UDCA to treat GDM; that it recruited a high number of participants; and that most of the participants were compliant with the study protocol. Importantly, there was no increase in adverse outcomes with the use of the drug, consistent with previous studies [22].

The primary endpoint of the GUARDS trial—maternal glycaemic control in the late third trimester—showed no significant differences between treatment groups. However, this sub-study suggests that UDCA may be associated with modest effects on maternal cardiac function. The use of a comprehensive echocardiographic protocol, with both conventional measures and advanced strain imaging, enabled detection of early and subtle alterations in maternal cardiovascular adaptation. In particular, left atrial strain indices, which are not very dependent on load and reflect atrioventricular coupling, appeared more sensitive to these changes, supporting their potential value for identifying early myocardial impairment in women with GDM.

However, several important limitations must be acknowledged. The effective sample size was relatively small, and the cardiac analyses were conducted as a per-protocol analysis with post-randomisation exclusion based on achieved UDCA concentrations, representing a deviation from the intention-to-treat principle and introducing potential selection bias. In addition, the trial was powered for glycaemic rather than cardiac endpoints and may therefore be underpowered to detect clinically meaningful differences in echocardiographic parameters.

The observed effects were modest and largely confined to selected left atrial functional indices, with no consistent effects on left ventricular systolic or diastolic function. Minimal clinically important differences for these indices in women with GDM are not established, and the clinical relevance of these small effect sizes remains uncertain. Furthermore, the evaluation of numerous echocardiographic parameters across multiple time points without adjustment for multiple tests increases the likelihood that some statistically significant results, especially those with borderline *p*-values, may represent chance findings rather than true treatment effects.

The postpartum follow-up period was relatively short, limiting conclusions regarding the persistence of the observed changes and their implications for long-term maternal cardiovascular risk. In addition, this was a single-centre study, which may limit its generalisability to other populations, healthcare settings, or ethnic groups. The study focused on subclinical echocardiographic markers rather than definitive cardiovascular endpoints; although such markers are sensitive indicators of early dysfunction, the absence of clinical outcome data precludes conclusions regarding true clinical or cardioprotective benefit.

## 5. Conclusions

Our study demonstrates that UDCA when given in mid-pregnancy to women with GDM has a small and time-dependent effect on left atrial function and myocardial performance in late pregnancy and postpartum. These findings have potential clinical and research implications, suggesting a potential new role for UDCA in cardiovascular protection during and after pregnancy. However, and given the absence of long-term follow-up and the lack of clinical cardiovascular endpoints, these findings should be interpreted with caution. Future large-scale, longitudinal studies are needed to validate these results, explore mechanisms of action, and determine whether UDCA can be integrated into routine care for pregnant women at risk of cardiovascular complications.

## Figures and Tables

**Table 1:** Baseline Participant Characteristics By Treatment Group.

Variable	Placebo (N = 57)	UDCA (N = 43)	*p* Value
Maternal age (years)	32.6 ± 6.1	34.5 ± 4.3	0.071
Body mass index (kg/m^2^)	29.3 ± 4.5	29.6 ± 3.9	0.765
White ethnicity	57/57 (100%)	42/43 (97.6%)	0.985
Nulliparity	25/57 (43.8%)	22/43 (51.1%)	0.601
Smoking	2/57 (3.5%)	5/43 (11.6%)	0.238
Assisted Reproductive Treatment	5/57 (8.7%)	2/43 (4.6%)	0.686

Values are presented as mean ± SD or n (%). Analyses are based on a per-protocol population for the UDCA group (n = 43) and the placebo group (n = 57).

**Table 2:** Comparison Of Cardiac Indices At Baseline Before Randomization.

Variable	Placebo (N = 57)	UDCA (N = 43)	*p* Value
**Left atrial size**			
Left atrial area (cm^2^)	14.82 (3.20)	14.40 (2.80)	0.490
Left atrial volume (mL)	59.48 (17.20)	61.70 (16.42)	0.514
Left atrial ejection fraction (%)	69.08 (6.51)	65.95 (10.45)	0.097
**Left atrial reservoir function**			
LASr_ED	50.74 (12.19)	47.88 (14.54)	0.281
LASr_AC			
**Left atrial conduit function**			
LAScd_ED	−33.77 (9.92)	−32.08 (11.33)	0.442
LAScd_AC	−28.95 (8.53)	−27.70 (9.75)	0.507
**Left atrial contractile function**			
LASct_ED			
LASct_AC	−14.20 (5.06)	−13.36 (5.41)	0.432
**Left ventricular systolic indices**			
Ejection fraction (%)	64.89 (10.48)	65.19 (6.84)	0.863
Global longitudinal systolic strain (%)	−19.80 (2.77)	−19.59 (2.85)	0.715

Values are mean (SD). Analyses are based on a per-protocol UDCA population (n = 43) and placebo group (n = 57). Abbreviations: LASr_ED, left atrial reservoir strain at end-diastole; LASr_AC, left atrial reservoir strain during atrial contraction; LAScd_ED, left atrial conduit strain at end-diastole; LAScd_AC, left atrial conduit strain during atrial contraction; LASct_ED, left atrial contractile strain at end-diastole; LASct_AC, left atrial contractile strain during atrial contraction.

**Table 3:** Comparison of cardiac indices in the third trimester and postpartum.

	36 Weeks’ Gestation			Postpartum		
Variable	Placebo (N = 54)	UDCA (N = 43)	*p* Value	Placebo (N = 48)	UDCA (N = 38)	*p* Value
**Left atrial (LA) reservoir function**						
LASr_ED	47.85 (13.16)	48.49 (16.42)	0.838	50.87 (13.54)	58.82 (19.90)	0.041
LASr_AC	41.15 (10.12)	40.45 (12.23)	0.767	43.55 (9.20)	49.28 (14.07)	0.036
**Left atrial conduit function**						
LAScd_ED	−32.16 (10.88)	−29.60 (12.86)	0.305	−35.05 (9.02)	−40.71 (14.65)	0.043
LAScd_AC	−26.03 (13.78)	−24.79 (10.37)	0.626	−30.37 (7.97)	−34.28 (11.30)	0.078
**Left atrial contractile function**						
LASct_ED	−15.70 (6.54)	−18.87 (6.60)	0.021	−15.87 (8.65)	−18.34 (9.24)	0.211
LASct_AC	−13.30 (4.89)	−15.65 (4.44)	0.016	−13.22 (6.41)	−15.00 (6.68)	0.221
**Left ventricular systolic index**						
Ejection fraction (%)	68.15 (8.89)	68.78 (8.87)	0.731	70.09 (8.33)	68.87 (8.23)	0.500

Values are mean (SD). Values represent unadjusted comparisons between groups at each time point. Comprehensive ventricular, haemodynamic, and right ventricular echocardiographic parameters are provided in Supplementary Tables S1B (36 weeks) and S1C (postpartum). Abbreviations: LASr_ED, left atrial reservoir strain at end-diastole; LASr_AC, left atrial reservoir strain during atrial contraction; LAScd_ED, left atrial conduit strain at end-diastole; LAScd_AC, left atrial conduit strain during atrial contraction; LASct_ED, left atrial contractile strain at end-diastole; LASct_AC, left atrial contractile strain during atrial contraction.

## Data Availability

Catalina De Paco Matallana served as the guarantor of this study, had full access to all of the data in the study, and accepts responsibility for the integrity of the data and the accuracy of the data analysis.

## Supplementary Materials

The following supporting information can be downloaded at https://www.mdpi.com/article/10.3390/jcm15020786/s1, Table S1. Baseline Echocardiographic Parameters Before Randomization. Table S2. Echocardiographic Parameters at 36 Weeks’ Gestation and Postpartum. Table S3. Longitudinal Mixed-Effects Model Results (Treatment × Time Interactions).
