# Supplementary material for: The Impact of Ursodeoxycholic Acid on Maternal Cardiac Function in Women with Gestational Diabetes Mellitus: A Randomized Controlled Study (GUARDS Trial)
Source: J Clin Med. 2026 Jan 19;15(2):786. doi: 10.3390/jcm15020786 (PMC12842236; doi:10.3390/jcm15020786)
Supplement: Supplementary file 1 [file jcm-15-00786-s001.zip › jcm-4066278-supplementary.pdf]

Table S1. Baseline Echocardiographic Parameters Before Randomization

Variable	Placebo (n = 57)	UDCA (n = 43)	P value
**Left ventricular diastolic function**			
Mitral valve E (cm/s)	83.70 (17.56)	85.62 (16.77)	0.581
Mitral valve A (cm/s)	56.56 (15.96)	60.59 (18.48)	0.255
Mitral valve E/A	1.53 (0.51)	1.57 (0.54)	0.719
Mitral valve E/e′	0.31 (0.08)	0.29 (0.07)	0.368
Isovolumic relaxation time (ms)	70.78 (16.09)	74.46 (12.86)	0.207
**Additional left ventricular systolic indices**			
Mitral valve s′ lateral (cm/s)	10.20 (2.06)	10.43 (2.28)	0.601
Mitral valve s′ septal (cm/s)	9.16 (1.69)	8.92 (1.27)	0.416
Isovolumic contraction time (ms)	66.94 (13.01)	65.25 (13.31)	0.554
Myocardial performance index	0.52 (0.09)	0.52 (0.09)	0.956
**Haemodynamics**			
Stroke volume (LVOT Doppler, mL)	77.47 (16.14)	73.95 (15.46)	0.271
Cardiac output (L/min)	6.22 (1.43)	5.99 (1.51)	0.436
Heart rate (bpm)	80.70 (10.90)	81.06 (8.80)	0.852
Structural cardiac indices			
Left ventricular mass (g)	152.26 (39.32)	148.40 (34.32)	0.604
Left ventricular posterior wall thickness in diastole (cm)	1.06 (0.23)	0.99 (0.16)	0.007
**Right ventricular functional indices**			
TAPSE (mm)	21.94 (3.87)	22.29 (4.09)	0.682
Fractional area change (%)	47.00 (5.65)	46.82 (5.64)	0.884
Tricuspid valve s′ (cm/s)	13.14 (2.51)	13.25 (2.27)	0.807

Values are mean (SD). These variables are provided for completeness and were not primary outcomes of the study. **Abbreviations**: E, early diastolic mitral inflow velocity; A, late diastolic mitral inflow velocity; E/A, ratio of early to late diastolic mitral inflow velocities; E/e′, ratio of early diastolic mitral inflow velocity to early diastolic mitral annular velocity; IVRT, isovolumic relaxation time; s′, systolic mitral or tricuspid annular velocity; GLS, global longitudinal systolic strain; LV, left ventricular; TAPSE, tricuspid annular plane systolic excursion; FAC, fractional area change; LVOT, left ventricular outflow tract.

Table S2. Echocardiographic Parameters at 36 Weeks' Gestation and Postpartum

	**36 weeks' gestation**			**Postpartum**		
**Variable**	**Placebo (N=54)**	**UDCA (N=43)**	**P value**	**Placebo (N=48)**	**UDCA (N=38)**	**P value**
**Left ventricular diastolic function**						
Mitral valve E(cm/sec)	82.48(18.12)	79.97(15.38)	0.401	83.67(17.54)	80.96(15.53)	0.450
Mitral valve A (cm/sec)	57.47(13.55)	60.67(19.70)	0.365	49.42(12.49)	51.51(14.29)	0.479
Mitral valve EA	1.49(0.51)	1.48(0.59)	0.921	1.75(0.73)	1.77(0.49)	0.852
Ee'	0.28(0.09)	0.28(0.07)	0.924	0.28(0.08)	0.29(0.08)	0.461
Isovolumic relaxation time (msec)	73.34(14.54)	71.76(15.31)	0.606	77.72(14.29)	77.86(16.18)	0.966
**Left atrial size**						
Left atrial area (cm^2)	15.52(2.97)	15.50(3.53)	0.981	13.73(3.11)	14.07(2.72)	0.598
Left atrial volume (ml)	59.74(18.08)	64.79(17.75)	0.173	54.45(20.98)	62.14(21.60)	0.103
Left atrial ejection fraction (%)	68.60(7.44)	69.35(7.61)	0.642	69.35(9.00)	67.10(9.15)	0.262
**Left ventricular systolic function**						
Mitral valve_s_lateral	10.14(2.27)	10.58(3.19)	0.447	9.75(2.25)	9.70(1.87)	0.919
Mitral valve_s_septal	9.04(1.47)	9.56(2.04)	0.161	8.16(1.31)	7.89(1.05)	0.309
Isovolumic contraction time (msec)	68.68(14.60)	66.76(13.64)	0.506	74.85(16.84)	72.55(15.72)	0.515
Global longitudinal strain (%)	-19.96(2.67)	-19.10(2.71)	0.126	-20.95(2.76)	-19.05(7.02)	0.127
Myocardial performance index	0.5650.09)	0.56(0.10)	0.779	0.54(0.10)	0.53(0.09)	0.492
**Hemodynamics**						
Stroke volume (ml)	74.50(15.32)	75.44(16.76)	0.775	80.75(17.51)	80.63(19.99)	0.977
Cardiac output (L/min)	6.09(1.09)	6.31(1.45)	0.356	5.64(1.32)	5.60(1.29)	0.892
Heart rate (bpm)	82.42(10.25)	84.51(11.21)	0.347	69.75(10.23)	71.09(10.49)	0.601
**Structural cardiac indices**						
Left ventricular mass (grams)	161.40(34.70)	169.23(37.58)	0.295	143.31(32.58)	146.72(31.28)	0.623
Left ventricular posterior wall thickness in diastole (cm)	1.11(0.20)	1.15(0.21)	0.332	1.03(0.18)	0.99(0.16)	0.359
**RV functional indices**						
TAPSE (mm)	21.28(3.68)	21.50(3.55)	0.787	20.35(3.90)	21.29(3.25)	0.246
Fractional area change (%)	47.12(6.33)	46.90(6.38)	0.875	46.03(5.56)	46.93(6.60)	0.524
Tricuspid Valve s (cm/sec)	12.89(2.62)	13.13(2.77)	0.661	12.38(1.94)	11.60(1.47)	0.037

Values are mean (SD). Values represent unadjusted comparisons between groups at each time point. **Abbreviations:** E, early diastolic mitral inflow velocity; A, late diastolic mitral inflow velocity; E/A, ratio of early to late diastolic mitral inflow velocities; E/e′, ratio of early diastolic mitral inflow velocity to early diastolic mitral annular velocity; IVRT, isovolumic relaxation time; s′, systolic mitral or tricuspid annular velocity; GLS, global longitudinal systolic strain; LV, left ventricular; TAPSE, tricuspid annular plane systolic excursion; FAC, fractional area change; LVOT, left ventricular outflow tract.

Table S3. Longitudinal Mixed-Effects Model Results (Treatment × Time Interactions)

Variable	Time point	β (Treatment ×Time)	95% CI	P value
LASct_AC	Late third trimester	−3.22	−6.06 to −0.37	0.031
LASct_AC	Postpartum	−1.14	−3.66 to 1.38	0.372
MV s′ septal	Late third trimester	0.75	0.01 to 1.48	0.039
MV s′ septal	Postpartum	0.06	−0.43 to 0.55	0.795
LASr_ED	Postpartum	10.69	2.90 to 18.48	0.007
LASr_AC	Postpartum	7.57	1.99 to 13.15	0.008
LAScd_ED	Postpartum	−7.03	−12.63 to −1.42	0.014
LASct_ED	Postpartum	−4.40	−8.23 to −0.57	0.002

β represents the fixed-effect coefficient for the **treatment × time interaction** from mixed-effects models (see Statistical Analysis). Late third trimester corresponds to the 36-week assessment.
Abbreviations: LASr_ED, left atrial reservoir strain at end-diastole; LASr_AC, left atrial reservoir strain during atrial contraction; LAScd_ED, left atrial conduit strain at end-diastole; LASct_ED, left atrial contractile strain at end-diastole; LASct_AC, left atrial contractile strain during atrial contraction; MV_s_septal, septal mitral annular systolic velocity.
